# Supplementary material for: Chemotaxis and degradation of organophosphate compound by a novel moderately thermo-halo tolerant Pseudomonas sp. strain BUR11: evidence for possible existence of two pathways for degradation
Source: PeerJ. 2015 Nov 10;3:e1378. doi: 10.7717/peerj.1378 (PMC4647611; doi:10.7717/peerj.1378)
Supplement: Supplemental Information 1 [file peerj-03-1378-s007.zip › Raw data for peerJ on demand/Raw data for HPLC chromatogram/par 0 h test.pdf]

SAMPLE INFORMATION

Sample Name:	par cont	Acquired By:	System
Sample Type:	Unknow n	Sample Set Name:	
Vial:	1	Acq. Method Set:	training
Injection #:	8	Processing Method:	par cont
Injection Volume:	20.00 ul	Channel Name:	486
Run Time:	15.0 Minutes	Proc. Chnl. Descr.:	
Date Acquired:	9/11/2013 5:21:19 PM IST		
Date Processed:	9/13/2013 1:02:26 PM IST		

Auto-Scaled Chromatogram

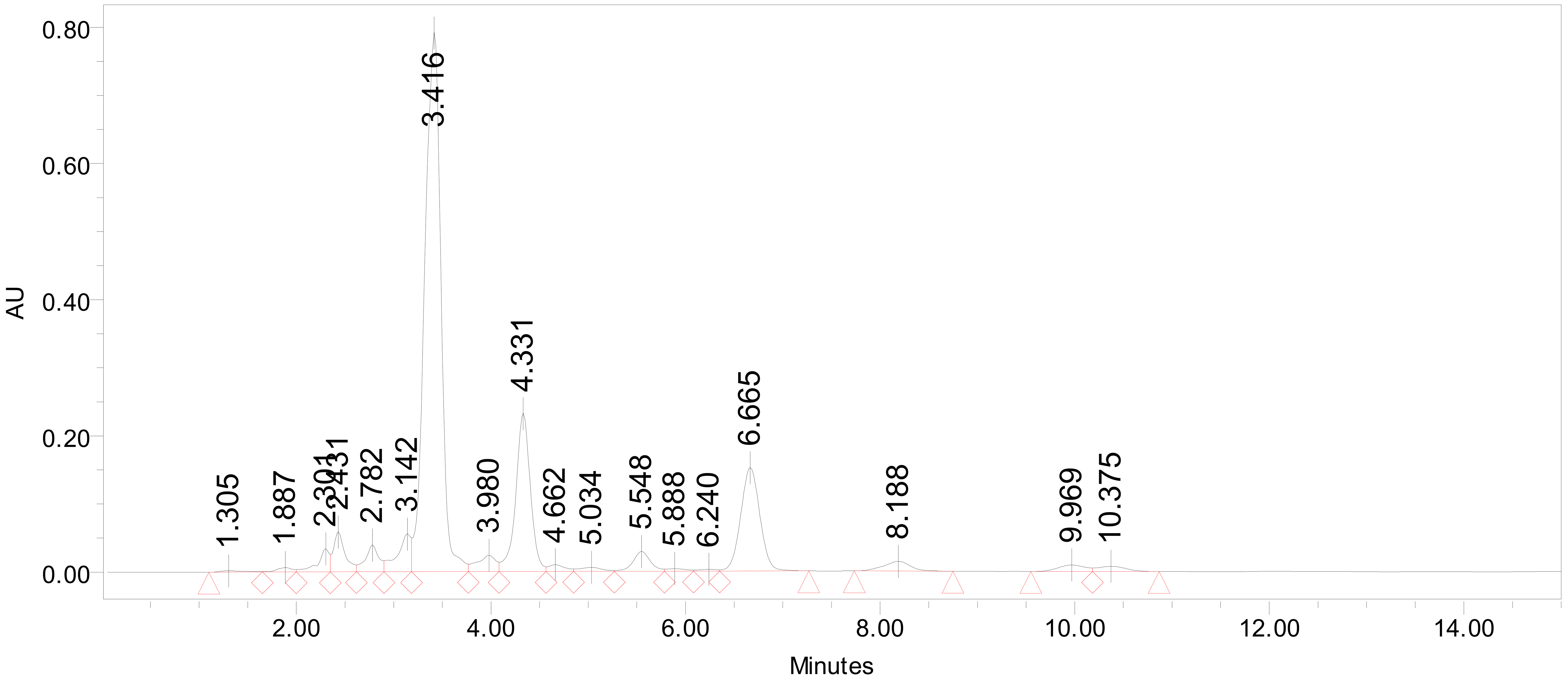

Peak Results

	Name	RT	Area	Height	Amount	Units
1		1.305	34621	1811		
2		1.887	72574	6469		
3		2.301	279383	33897		
4		2.431	481754	58606		
5		2.782	377943	39245		
6		3.142	530923	55814		
7		3.416	9162348	791680		
8		3.980	318735	23959		
9		4.331	2435391	232430		
10		4.662	117316	9818		

Peak Results

	Name	RT	Area	Height	Amount	Units
11		5.034	94403	5805		
12		5.548	357720	29000		
13		5.888	51758	3736		
14		6.240	34527	2591		
15		6.665	2073915	152014		
16		8.188	278197	14394		
17		9.969	185788	9568		
18		10.375	155049	7617		
