# Supplementary material for: Chemotaxis and degradation of organophosphate compound by a novel moderately thermo-halo tolerant Pseudomonas sp. strain BUR11: evidence for possible existence of two pathways for degradation
Source: PeerJ. 2015 Nov 10;3:e1378. doi: 10.7717/peerj.1378 (PMC4647611; doi:10.7717/peerj.1378)
Supplement: Supplemental Information 1 [file peerj-03-1378-s007.zip › Raw data for peerJ on demand/Raw data for HPLC chromatogram/par 24 h test.pdf]

SAMPLE INFORMATION

Sample Name:	par 24 t	Acquired By:	System
Sample Type:	Unknow n	Sample Set Name:	
Vial:	1	Acq. Method Set:	training
Injection #:	3	Processing Method:	par 24 t
Injection Volume:	20.00 ul	Channel Name:	486
Run Time:	15.0 Minutes	Proc. Chnl. Descr.:	
Date Acquired:	9/12/2013 1:06:56 PM IST		
Date Processed:	9/13/2013 1:05:11 PM IST		

Auto-Scaled Chromatogram

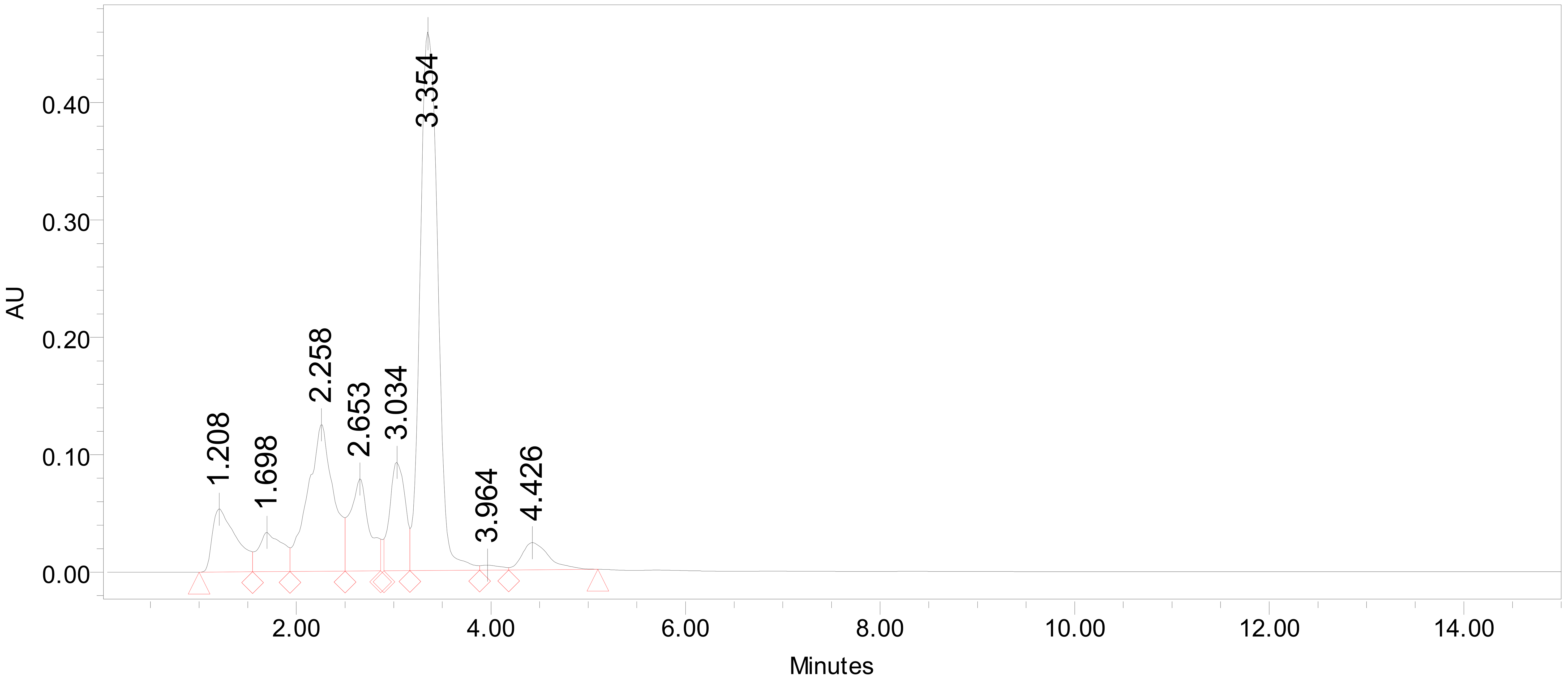

Peak Results

	Name	RT	Area	Height	Amount	Units
1		1.208	915590	53684		
2		1.698	581744	33327		
3		2.258	2312866	125280		
4		2.653	1081953	78429		
5		3.034	1020487	92423		
6		3.354	5824878	459393		
7		3.964	60086	4276		
8		4.426	447894	23247		
