# Supplementary material for: Chemotaxis and degradation of organophosphate compound by a novel moderately thermo-halo tolerant Pseudomonas sp. strain BUR11: evidence for possible existence of two pathways for degradation
Source: PeerJ. 2015 Nov 10;3:e1378. doi: 10.7717/peerj.1378 (PMC4647611; doi:10.7717/peerj.1378)
Supplement: Supplemental Information 1 [file peerj-03-1378-s007.zip › Raw data for peerJ on demand/Raw data for HPLC chromatogram/par 48 h test.pdf]

SAMPLE INFORMATION

Sample Name:	par 72 t	Acquired By:	System
Sample Type:	Unknow n	Sample Set Name:	
Vial:	1	Acq. Method Set:	training
Injection #:	5	Processing Method:	par 48 t
Injection Volume:	20.00 ul	Channel Name:	486
Run Time:	15.0 Minutes	Proc. Chnl. Descr.:	
Date Acquired:	9/12/2013 2:06:09 PM IST		
Date Processed:	9/13/2013 1:07:31 PM IST		

Auto-Scaled Chromatogram

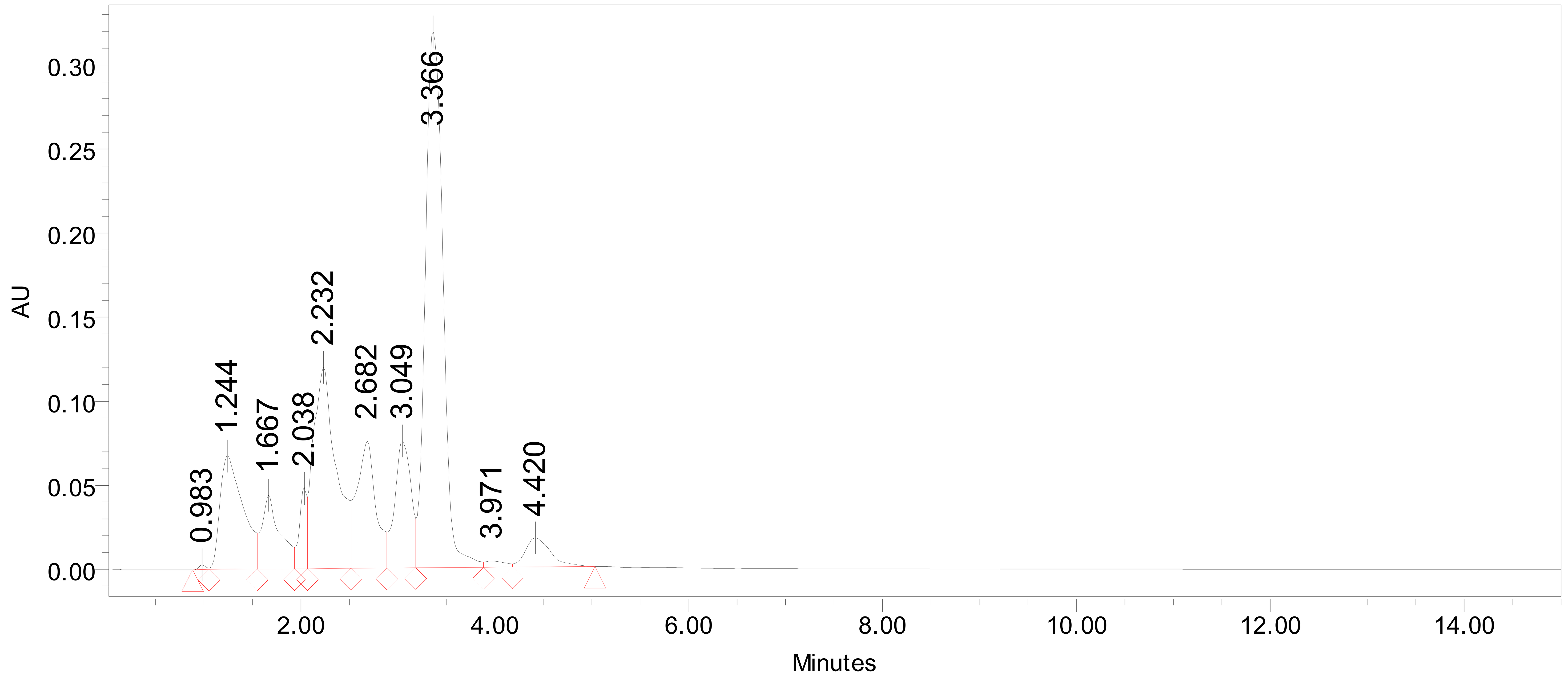

Peak Results

	Name	RT	Area	Height	Amount	Units
1		0.983	12985	2762		
2		1.244	1111830	67503		
3		1.667	576424	43810		
4		2.038	257508	48246		
5		2.232	1972394	119882		
6		2.682	1031369	75587		
7		3.049	881003	75494		
8		3.366	4051921	318920		
9		3.971	54199	3746		
10		4.420	312672	17266		
