# Supplementary material for: Chemotaxis and degradation of organophosphate compound by a novel moderately thermo-halo tolerant Pseudomonas sp. strain BUR11: evidence for possible existence of two pathways for degradation
Source: PeerJ. 2015 Nov 10;3:e1378. doi: 10.7717/peerj.1378 (PMC4647611; doi:10.7717/peerj.1378)
Supplement: Supplemental Information 1 [file peerj-03-1378-s007.zip › Raw data for peerJ on demand/Raw data for HPLC chromatogram/par 72 h test.pdf]

C I F Bose Institute

SAMPLE INFORMATION

Sample Name:	par 48 t	Acquired By:	System
Sample Type:	Unknow n	Sample Set Name:	
Vial:	1	Acq. Method Set:	training
Injection #:	4	Processing Method:	par 72 t
Injection Volume:	20.00 ul	Channel Name:	486
Run Time:	15.0 Minutes	Proc. Chnl. Descr.:	
Date Acquired:	9/12/2013 1:38:56 PM IST		
Date Processed:	9/13/2013 1:08:37 PM IST		

Auto-Scaled Chromatogram

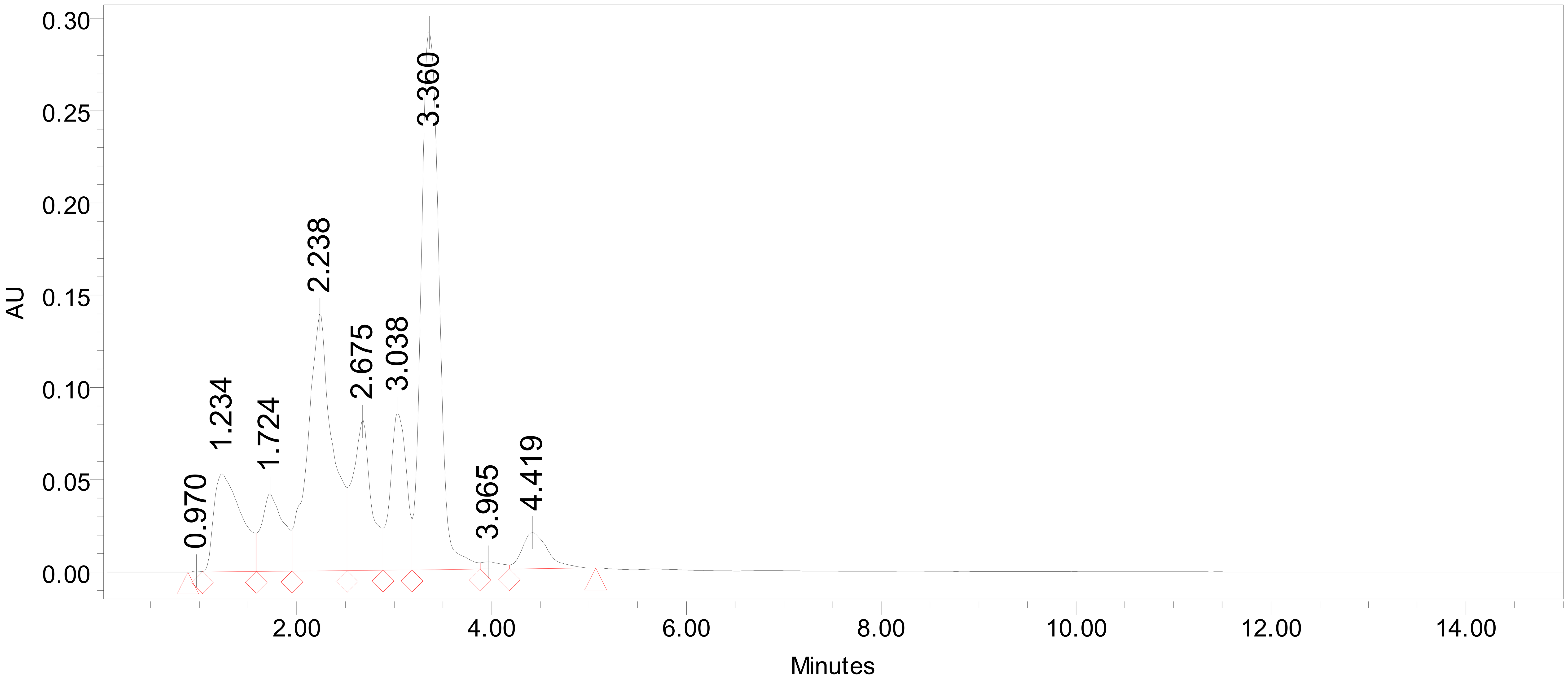

Peak Results

	Name	RT	Area	Height	Amount	Units
1		0.970	2974	638		
2		1.234	1045735	53118		
3		1.724	666354	42191		
4		2.238	2469610	139122		
5		2.675	1084261	81501		
6		3.038	982502	85441		
7		3.360	3732910	293001		
8		3.965	57397	3953		
9		4.419	360011	19602		
