# Supplementary material for: Chemotaxis and degradation of organophosphate compound by a novel moderately thermo-halo tolerant Pseudomonas sp. strain BUR11: evidence for possible existence of two pathways for degradation
Source: PeerJ. 2015 Nov 10;3:e1378. doi: 10.7717/peerj.1378 (PMC4647611; doi:10.7717/peerj.1378)
Supplement: Supplemental Information 1 [file peerj-03-1378-s007.zip › Raw data for peerJ on demand/Raw data for HPLC chromatogram/par 96 h test.pdf]

SAMPLE INFORMATION

Sample Name:	par 96 t	Acquired By:	System
Sample Type:	Unknow n	Sample Set Name:	
Vial:	1	Acq. Method Set:	training
Injection #:	7	Processing Method:	par 96 t
Injection Volume:	20.00 ul	Channel Name:	486
Run Time:	15.0 Minutes	Proc. Chnl. Descr.:	
Date Acquired:	9/12/2013 3:15:09 PM IST		
Date Processed:	9/13/2013 1:09:48 PM IST		

Auto-Scaled Chromatogram

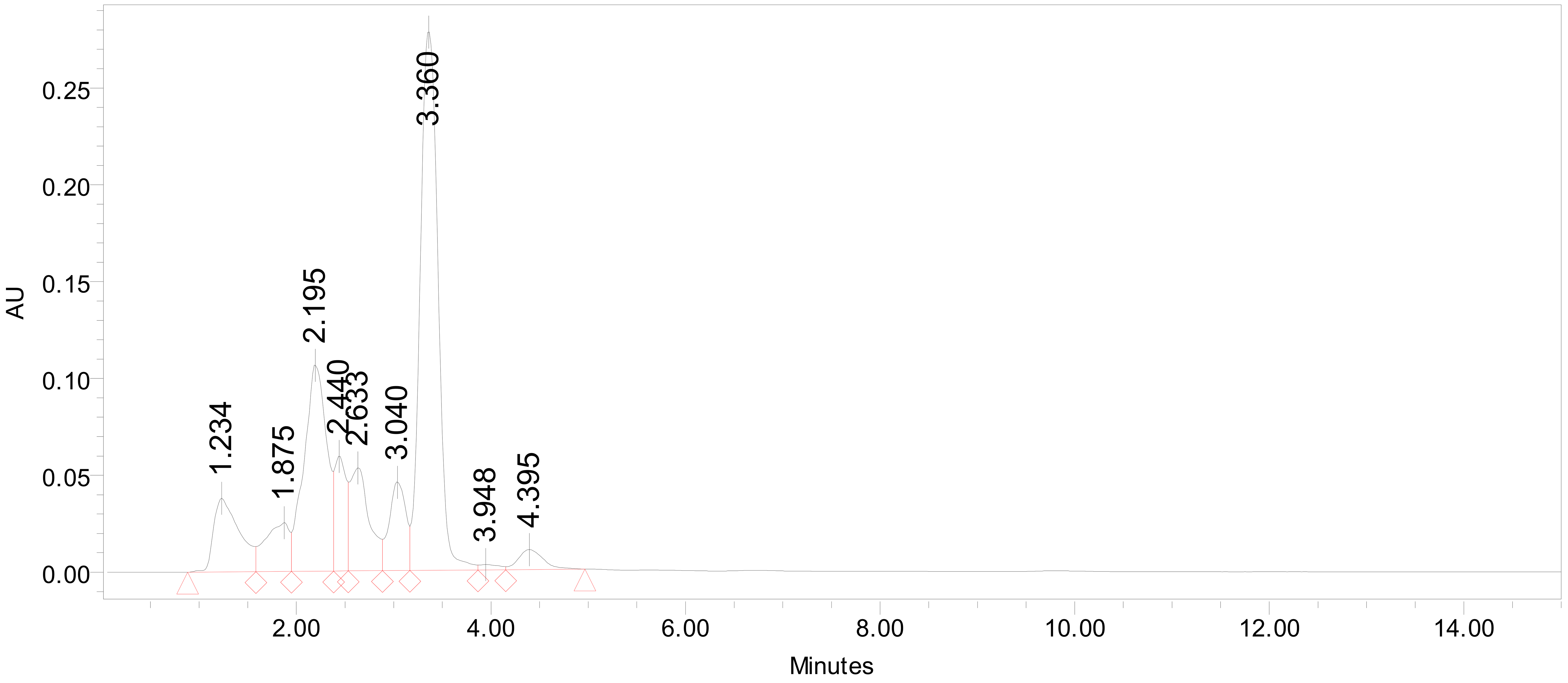

Peak Results

	Name	RT	Area	Height	Amount	Units
1		1.234	703346	37959		
2		1.875	438437	25282		
3		2.195	1752517	107255		
4		2.440	484047	59246		
5		2.633	740317	53165		
6		3.040	546469	45900		
7		3.360	3548204	278711		
8		3.948	39336	2716		
9		4.395	184959	10301		
