# Supplementary material for: Chemotaxis and degradation of organophosphate compound by a novel moderately thermo-halo tolerant Pseudomonas sp. strain BUR11: evidence for possible existence of two pathways for degradation
Source: PeerJ. 2015 Nov 10;3:e1378. doi: 10.7717/peerj.1378 (PMC4647611; doi:10.7717/peerj.1378)
Supplement: Supplemental Information 1 [file peerj-03-1378-s007.zip › Raw data for peerJ on demand/Raw data of GC for evidence of presence of Aminoparathion in the extract sample/For Figure S5 GC-Screening.pdf]

GC

File :D:\GC-MS-Year-2015\JAN-15\06-01-15\7814-265-0059.D
Operator : PRABAKARAN
Acquired : 6 Jan 2015 11:01 using AcqMethod UN_KNOWN_WITHOUT FORNT_COLUMN.M
Instrument : GC MS
Sample Name: SAMPLE- I
Misc Info :
Vial Number: 15

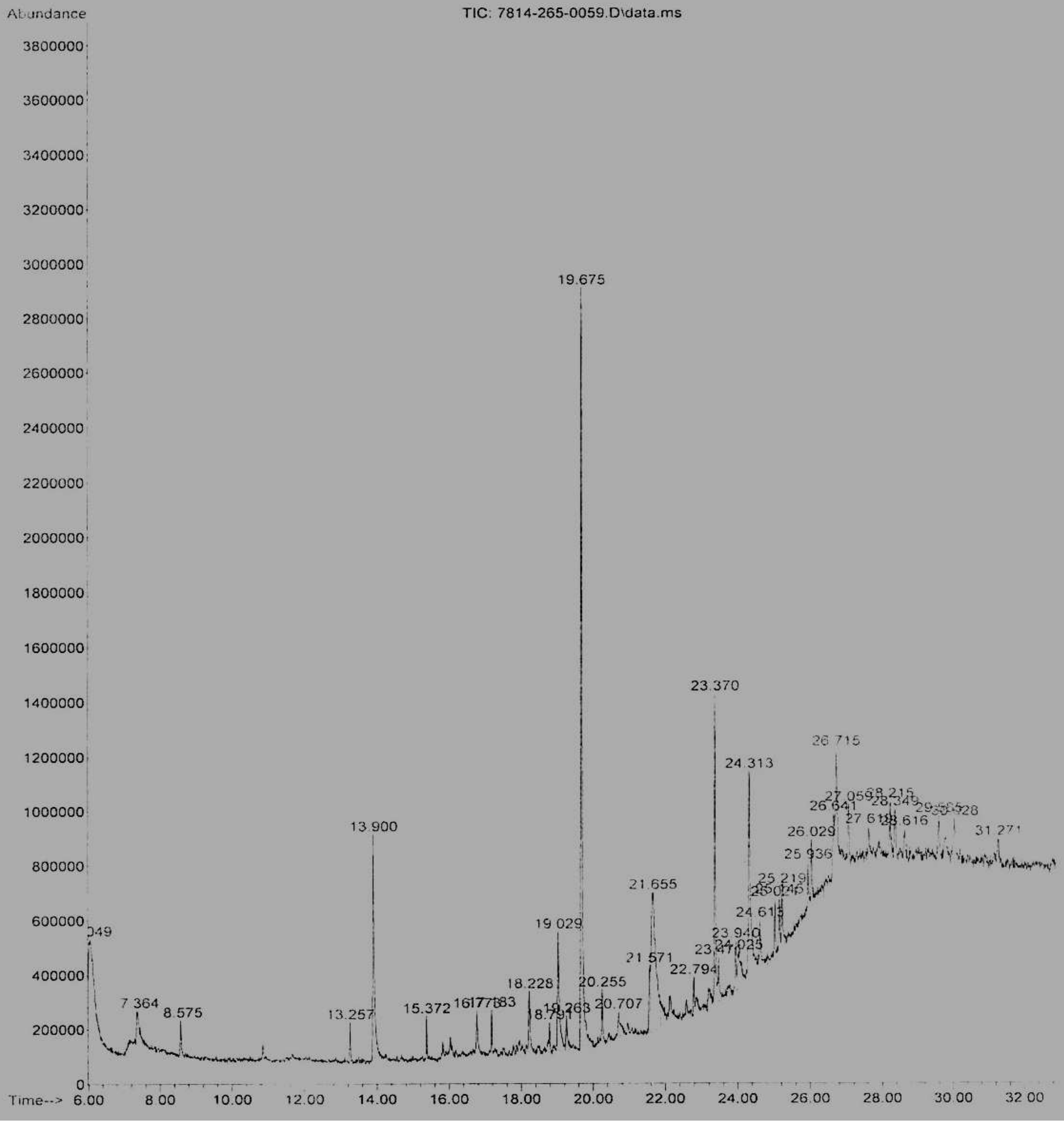

BVCPS-INDIA PVT.LTD,CHENNAI Library Search Report

Data Path : D:\GC-MS-Year-2015\JAN-15\06-01-15\
Data File : 7814-265-0059.D
Acq On : 6 Jan 2015 11:01
Operator : PRABAKARAN
Sample : SAMPLE- I
Misc :
ALS Vial : 15 Sample Multiplier: 1

Search Libraries: C:\Database\NIST11.L Minimum Quality: 0

Unknown Spectrum: Apex
Integration Events: ChemStation Integrator - 7814-357-0048.E

Pk#	RT	Area%	Library/ID	Ref#	CAS#	Qual
1	6.052	2.37	C:\Database\NIST11.L			
			Adipamide	20534	000628-94-4	49
			Pregn-5-en-3-ol, 20-amino-, (3.beta.,20S)-	158939	005035-10-9	47
			2,2-Dichlorocyclopropanecarboxamid	26258	075885-60-8	47
2	7.367	0.79	C:\Database\NIST11.L			
			Atrolactic acid	35611	000515-30-0	59
			.alpha.-Hydroxy-.alpha.-methylbenzyl phenyl ketone	82925	005623-26-7	59
			Benzaldehyde dimethyl acetal	26004	001125-88-8	59
3	8.578	0.56	C:\Database\NIST11.L			
			O,O,O-Triethyl thiophosphate	59987	000126-68-1	94
			O,O,O-Triethyl thiophosphate	59986	000126-68-1	91
			Asulam	86364	003337-71-1	43
4	13.258	0.60	C:\Database\NIST11.L			
			Cycloheptasiloxane, tetradecamethyl-	236968	000107-50-6	91
			Cycloheptasiloxane, tetradecamethyl-	236969	000107-50-6	91
			Pentasiloxane, dodecamethyl-	204093	000141-63-9	38
5	13.897	4.62	C:\Database\NIST11.L			
			Butylated Hydroxytoluene	77554	000128-37-0	97
			Butylated Hydroxytoluene	77555	000128-37-0	97
			Butylated Hydroxytoluene	77553	000128-37-0	91
6	15.375	0.51	C:\Database\NIST11.L			
			Benzoic acid, 2,4-bis[(trimethylsilyl)oxy]-, trimethylsilyl ester	196465	010586-16-0	38
			Silane, [[4-[1,2-bis[(trimethylsilyl)oxy]ethyl]-1,2-phenylene]bis(oxy)]bis[trimethyl-	228735	056114-62-6	37
			Cyclopentasiloxane, decamethyl-	196318	000541-02-6	35
7	16.772	0.86	C:\Database\NIST11.L			
			Phenol, 2,6-bis(1-methylpropyl)-	66102	005510-99-6	50
			Bis(2-methylpropyl) 4,5-dimethylbenzene-1,2-dicarboxylate	149718	1000305-39-8	50
			3-Buten-2-one, 4-(2,6,6-trimethyl-1-cyclohexen-1-yl)-	54652	014901-07-6	50
8	17.180	0.47	C:\Database\NIST11.L			
			1,1,1,5,7,7,7-Heptamethyl-3,3-bis(trimethylsiloxy)tetrasiloxane	225661	038147-00-1	45
			Hexasiloxane, tetradecamethyl-	228692	000107-52-8	27
			2H-1,4-Benzodiazepin-2-one, 7-chloro-1,3-dihydro-5-phenyl-1-(trimethylsilyl)-3-[(trimethylsilyl)oxy]-	222094	055319-93-2	22
9	18.228	1.26	C:\Database\NIST11.L			
			Dibutyl phthalate	125787	000084-74-2	64
			1,2-Benzenedicarboxylic acid, dihe	172559	000084-75-3	59

```
                 xyl ester
                 Phthalic acid, heptyl hept-4-yl es 191990 1000356-78-8 53
                 ter

10  18.792  0.61 C:\Database\NIST11.L
                 Octasiloxane, 1,1,3,3,5,5,7,7,9,9, 240341 019095-24-0 40
                 11,11,13,13,15,15-hexadecamethyl-
                 Cyclononasiloxane, octadecamethyl- 242431 000556-71-8 32
                 1,1,1,5,7,7,7-Heptamethyl-3,3-bis( 225661 038147-00-1 32
                 trimethylsiloxy)tetrasiloxane

11  19.030  3.45 C:\Database\NIST11.L
                 Aminoparathion                     111167 003735-01-1 94
                 4-Aminothiophenol                   10811 001193-02-8 25
                 3-Aminobenzenethiol                 10813 022948-02-3 22

12  19.260  0.81 C:\Database\NIST11.L
                 Dibutyl phthalate                  125786 000084-74-2 46
                 Tricyclo[4.3.1.1(3,8)]undecane-3-c  67770 031061-61-7 46
                 arboxylic acid, methyl ester
                 m-Tolyl isothiocyanate              23555 000621-30-7 46

13  19.676 14.80 C:\Database\NIST11.L
                 Parathion                          136570 000056-38-2 96
                 Parathion                          136574 000056-38-2 96
                 Parathion                          136573 000056-38-2 95

14  20.256  0.89 C:\Database\NIST11.L
                 Dodecanoic acid, 3,7,11-trimethyl- 234094 1000313-98-8 37
                 3,4-bis(trimethylsilyloxy)-,trimet
                 hylsilyl ester
                 Sarcosine, N-isobutyryl-, tetradec 187376 1000321-27-5 22
                 yl ester
                 Terephthalic acid, but-2-enyl hept 159716 1000356-31-5 22
                 yl ester

15  20.709  0.88 C:\Database\NIST11.L
                 l-Alanine, N-(5-chlorovaleryl)-, m  78470 1000299-71-3 38
                 ethyl ester
                 2,2-Dichlorocyclopropanecarboxamid  26258 075885-60-8 38
                 Metaraminol                         36216 000054-49-9 37

16  21.570  1.19 C:\Database\NIST11.L
                 Benzaldehyde, 2-nitro-, diaminomet  67108 102632-31-5 43
                 hylidenhydrazone
                 Sarcosine, N-isobutyryl-, tetradec 187376 1000321-27-5 38
                 yl ester
                 Octasiloxane, 1,1,3,3,5,5,7,7,9,9, 240341 019095-24-0 38
                 11,11,13,13,15,15-hexadecamethyl-

17  21.652 10.07 C:\Database\NIST11.L
                 Phenol, 4,4'-(1-methylethylidene)b  84554 000080-05-7 98
                 is-
                 Phenol, 4,4'-(1-methylethylidene)b  84556 000080-05-7 95
                 is-
                 Phenol, 4,4'-(1-methylethylidene)b  84555 000080-05-7 70
                 is-

18  22.796  0.63 C:\Database\NIST11.L
                 Norpseudoephedrine                  24943 036393-56-3 30
                 Phenethylamine, p,.alpha.-dimethyl  23388 000064-11-9 27
                 1-Methyl-2-phenoxyethylamine        24974 035205-54-0 27

19  23.368  4.72 C:\Database\NIST11.L
                 Hexanedioic acid, bis(2-ethylhexyl 196965 000103-23-1 93
                 ) ester
                 Diisooctyl adipate                 196853 001330-86-5 90
                 Hexanedioic acid, bis(2-ethylhexyl 196973 000103-23-1 90
                 ) ester

20  23.472  0.91 C:\Database\NIST11.L
                 1,4-Benzenedicarboxamide, N,N'-bis 222756 068516-51-8 27
```

			(2-hydroxy-1-methyl-2-phenylethyl)			
			Cyclotrisiloxane, hexamethyl-	79619	000541-05-9	27
			5-Acetamido-4,7-dioxo-4,7-dihydrob	67100	153136-27-7	27
			enzofurazan			
21	23.940	0.77	C:\Database\NIST11.L			
			Octasiloxane, 1,1,3,3,5,5,7,7,9,9,	240341	019095-24-0	64
			11,11,13,13,15,15-hexadecamethyl-			
			Cyclononasiloxane, octadecamethyl-	242431	000556-71-8	37
			2-Ethylacridine	66996	055751-83-2	25
22	24.022	1.36	C:\Database\NIST11.L			
			Bifemelane	118352	090293-01-9	43
			Propanamide	727	000079-05-0	27
			Epinephrine	48246	000051-43-4	25
23	24.312	6.93	C:\Database\NIST11.L			
			Cyclohexa-2,5-diene-1,4-dione, 2-m	66749	002158-89-6	44
			ethyl-5-(4-morpholinyl)-			
			Benzo[h]quinoline, 2,4-dimethyl-	67018	000605-67-4	38
			2-Ethylacridine	66996	055751-83-2	30
24	24.616	1.35	C:\Database\NIST11.L			
			Naphthalene, 6-chloro-1-nitro-	66603	038396-29-1	47
			1H-Indole-3-ethanamine, 6-fluoro-.	54300	077590-52-4	38
			beta.-methyl-			
			Propanamide	727	000079-05-0	35
25	25.025	1.38	C:\Database\NIST11.L			
			Octasiloxane, 1,1,3,3,5,5,7,7,9,9,	240341	019095-24-0	83
			11,11,13,13,15,15-hexadecamethyl-			
			Tetrasiloxane, decamethyl-	152279	000141-62-8	43
			2-Ethylacridine	66996	055751-83-2	38
26	25.144	1.62	C:\Database\NIST11.L			
			Eicosane	129490	000112-95-8	40
			Eicosane	129491	000112-95-8	40
			Benzo[h]quinoline, 2,4-dimethyl-	67018	000605-67-4	30
27	25.218	1.61	C:\Database\NIST11.L			
			Indolizine, 2-(4-methylphenyl)-	67014	007496-81-3	38
			4-Quinolinecarboxylic acid, 2-chlo	66606	005467-57-2	38
			ro-			
			Tetrasiloxane, decamethyl-	152278	000141-62-8	35
28	25.939	2.36	C:\Database\NIST11.L			
			Benzo[h]quinoline, 2,4-dimethyl-	67018	000605-67-4	52
			Cyclotrisiloxane, hexamethyl-	79619	000541-05-9	50
			Tetrasiloxane, decamethyl-	152279	000141-62-8	50
29	26.028	2.27	C:\Database\NIST11.L			
			Octasiloxane, 1,1,3,3,5,5,7,7,9,9,	240341	019095-24-0	38
			11,11,13,13,15,15-hexadecamethyl-			
			Benzo[h]quinoline, 2,4-dimethyl-	67018	000605-67-4	35
			Cyclotrisiloxane, hexamethyl-	79619	000541-05-9	35
30	26.644	3.38	C:\Database\NIST11.L			
			Tetrasiloxane, decamethyl-	152278	000141-62-8	43
			9H-Fluorene-2-carboxylic acid, 9-o	128371	1000316-02-1	35
			xo-, (2-hydroxyethyl)(methyl)amide			
			Cyclotrisiloxane, hexamethyl-	79617	000541-05-9	30
31	26.719	6.19	C:\Database\NIST11.L			
			Benzo[h]quinoline, 2,4-dimethyl-	67018	000605-67-4	43
			Cyclotrisiloxane, hexamethyl-	79619	000541-05-9	43
			Cyclotrisiloxane, hexamethyl-	79617	000541-05-9	30
32	27.060	3.50	C:\Database\NIST11.L			
			1,2-Bis(trimethylsilyl)benzene	78918	017151-09-6	50
			Octasiloxane, 1,1,3,3,5,5,7,7,9,9,	240341	019095-24-0	45
			11,11,13,13,15,15-hexadecamethyl-			
			Tris(tert-butyldimethylsilyloxy)ar	230548	1000366-57-5	43

```
                  sane

 33  27.617  4.46 C:\Database\NIST11.L
                  Benzo[h]quinoline, 2,4-dimethyl-     67018 000605-67-4 43
                  Cyclotrisiloxane, hexamethyl-        79619 000541-05-9 43
                  1,4-Bis(trimethylsilyl)benzene       78919 013183-70-5 43

 34  28.212  3.10 C:\Database\NIST11.L
                  Cyclotrisiloxane, hexamethyl-        79617 000541-05-9 49
                  1,2-Bis(trimethylsilyl)benzene       78918 017151-09-6 49
                  Tris(tert-butyldimethylsilyloxy)ar 230548 1000366-57-5 46
                  sane

 35  28.345  2.98 C:\Database\NIST11.L
                  Silicic acid, diethyl bis(trimethy 140472 003555-45-1 38
                  lsilyl) ester
                  2,4,6-Cycloheptatrien-1-one, 3,5-b 102104 1000161-21-8 38
                  is-trimethylsilyl-
                  Benzestrol di-TMS derivative       225367 1000137-02-9 37

 36  28.613  2.63 C:\Database\NIST11.L
                  Cyclotrisiloxane, hexamethyl-        79619 000541-05-9 50
                  Benzo[h]quinoline, 2,4-dimethyl-     67018 000605-67-4 50
                  Tetrasiloxane, decamethyl-          152276 000141-62-8 47

 37  29.586  1.36 C:\Database\NIST11.L
                  9H-Fluorene-2-carboxylic acid, 9-o 128371 1000316-02-5 47
                  xo-, (2-hydroxyethyl)(methyl)amide
                  Tetrasiloxane, decamethyl-          152279 000141-62-8 43
                  Cyclotrisiloxane, hexamethyl-        79619 000541-05-9 43

 38  30.024  1.03 C:\Database\NIST11.L
                  Acetamide, N-[4-(trimethylsilyl)ph  66793 017983-71-0 53
                  enyl]-
                  (E)-2-bromobutyloxychalcone         189228 1000370-38-6 47
                  1,2-Bis(trimethylsilyl)benzene       78918 017151-09-6 47

 39  31.272  0.69 C:\Database\NIST11.L
                  Methyl 3-bromo-1-adamantaneacetate 132323 014575-01-0 50
                  Cyclotrisiloxane, hexamethyl-        79617 000541-05-9 46
                  Tris(tert-butyldimethylsilyloxy)ar 230548 1000366-57-5 46
                  sane
```
