# Supplementary material for: Chemotaxis and degradation of organophosphate compound by a novel moderately thermo-halo tolerant Pseudomonas sp. strain BUR11: evidence for possible existence of two pathways for degradation
Source: PeerJ. 2015 Nov 10;3:e1378. doi: 10.7717/peerj.1378 (PMC4647611; doi:10.7717/peerj.1378)
Supplement: Supplemental Information 1 [file peerj-03-1378-s007.zip › Raw data for peerJ on demand/Raw data for standard curve/Standard curve.doc]

**For Figure 4A and 4B (Raw data on the basis of HPLC analysis)**

1. **Standard curve for parathion quantification**

| **Concentration (mg L^-1^)** | **Area** |
| --- | --- |
| 0 | 0 |
| 25 | 1338681 |
| 50 | 3131327 |
| 250 | 13243568 |
| 500 | 26866637 |

2. **Parathion degradation with time**

| **Time (h)** | **Area** | **Concentration (mg L^-1^)** | **Percentage of degradation (%)** |
| --- | --- | --- | --- |
| 0 | 9162348 | 170 | 0 |
| 24 | 5824878 | 110 | 35.3 |
| 48 | 4051921 | 75 | 55.88 |
| 72 | 3732910 | 69 | 59.4 |
| 96 | 3548204 | 54 | 61.6 |

3. 4-nitrophenol standard curve

| **Concentration (mg L^-1^)** | **Area** |
| --- | --- |
| 0 | 0 |
| 250 | 7794752 |
| 500 | 15359956 |

4. **Quantification of 4-nitrophenol with time from standard curve**

| **Time (h)** | **Area** | **4-nitrophenol (mg L^-1^)** |
| --- | --- | --- |
| 0 | 0 | 0 |
| 24 | 1080953 | 30 |
| 48 | 1031369 | 29 |
| 72 | 1084261 | 30.2 |
| 96 | 740317 | 15 |

5. **Concentration of other hydrolytic intermediates** (the abundance of these peaks (mentioned compounds) are very less and it is very difficult to calculate their concentration from standard curve. Thus concentrations were calculated from known concentration of respective standard compounds by using the following fomula- **Concentration of unkown (mg/L) = Area of unknown / Area of known X concentration of known (mg/L**)

| **Time (h)** | **Area** | **Parabenzoquinone (mg L^-1^)** | **Area** | **Hydroquinone (mg L^-1^)** | **Area** | **1,2,4 benzenetriol (mg L^-1^)** |
| --- | --- | --- | --- | --- | --- | --- |
| 0 | 0 | 0 | 0 | 0 | 0 | 0 |
| 24 | 982502 | 1.3 | 2312866 | 0.84 | 581744 | 0.61 |
| 48 | 1020487 | 1.5 | 1972394 | 0.72 | 257508 | 0.272 |
| 72 | 881003 | 0.72 | 484047 | 0.72 | 2469610 | 2.61 |
| 96 | 546469 | 0.27 | 284047 | 0.176 | 1752517 | 1.85 |
